# Lack of association between serum vitamin B12 and nocturnal sleep parameters following cyanocobalamin supplementation in healthy adults

**DOI:** 10.1016/j.heliyon.2022.e08831

**Published:** 2022-01-25

**Authors:** Toshiba Channer-Wallen, Paula Dawson, Peta-Gaye Thomas-Brown, Maxine Gossell-Williams

**Affiliations:** a Caribbean Institute for Health Research (CAIHR), The University of the West Indies, Kingston 7, Jamaica; b Department of Surgery, Radiology, Anaesthesia and Intensive Care & Emergency Medicine, Faculty of Medical Sciences, The University of the West Indies, Kingston 7, Jamaica; c Section of Pharmacology and Pharmacy, Faculty of Medical Sciences, The University of the West Indies, Kingston 7, Jamaica; d School of Pharmacy, College of Health Sciences, The University of Technology, 237 Old Hope Road, Kingston 6, Jamaica

**Keywords:** Vitamin B12, Cyanocobalamin, Sleep parameters, Actigraphy

## Abstract

**Background:**

This study examined the effect of vitamin B12 supplementation on sleep parameters of latency, total sleep time, wake-after-sleep onset, total time in bed and sleep efficiency in healthy adults.

**Methods:**

This quasi-experimental pre-test, post-test design recruited adults 25–50 years old, with normal vitamin B12 levels. Sleep parameters were assessed using Phillips Respironics Actiwatch-2® on non-dominant wrist from Monday to Thursday for four weeks. Pre-supplementation data was collected for the first two weeks; then participants started 3mg pre-packaged cyanocobalamin supplements daily for 14 days. Post-supplementation data was collected for weeks three and four. Serum was collected by venipuncture at the beginning and end of the study for vitamin B12 assay. Descriptive statistics involved median and interquartile range [IQR]. A comparison of the sleep parameters before and after cyanocobalamin supplementation was done using non-parametric inferential analysis.

**Results:**

Fourteen healthy adult participants completed the study; nine females and five males with median age of 37[17] years and a normal range of serum vitamin B12 level (169–695 pmol/L). Median serum vitamin B12 level was significantly elevated following supplementation (355[217] to 961[679]) pmol/L; p = 0.020); but there was no change in any of the sleep parameters measured. Spearman's rho correlation analysis showed no correlation between serum vitamin B12 levels and the sleep parameters for pre-supplementation and post-supplementation weeks.

**Conclusion:**

Two weeks of cyanocobalamin supplementation (3mg/day) resulted in the expected increase in serum vitamin B12 levels in healthy adults but did not influence their sleep wake activity.

## 1 Introduction

The relationship between insufficient sleep and diseases has increased the interest in the role of diet and nutrition in sleep. Several studies that assessed sleep quality and sleep-wake disorders using sleep diaries or questionnaires have reported contradictory associations with vitamin B12 (cobalamin) [1, 2, 3]. The National Health and Nutrition Examination Surveys (NHANES) study, which was cross-sectional surveys of health and nutritional status of the civilian population of the United States of America is one of the largest studies with available serum vitamin B12 concentrations. Using data from 2459 adults of both genders between age 20–85 years, it was concluded that vitamin B12 levels were inversely associated with sleep duration [4]. In another study among 355 female students in Saudi Arabia, using the PSQI questionnaire, the association between sleep duration and serum vitamin B12 status varied based on whether participants had low (<221 pmol/L), normal (221–701 pmol/L) or excess (>701 pmol/L) concentrations. However, after controlling for confounders including stress and waist hip ratio (WHR), only 22 participants with excess vitamin B12 showed a shorter sleep latency [5]. A search of the literature only identified one vitamin B12 supplementation study where sleep was measured using actigraphy (activity-base sleep-wake monitoring) and involved administering 3mg/day of vitamin B12 in 14 healthy male participants [6]. All participants showed increase serum vitamin B12 levels with 14 days of supplementation, and it was determined that there was increased activity during nocturnal sleep, measured as movements per hour during the period of 11:00pm to 7:00am.

Actigraphy is now considered a validated method to measure sleep-wake activity in adults [7, 8] and current instruments provide algorithms to calculate additional measures for nocturnal sleep activity. These include total sleep time (the total minutes of sleep recorded in a complete episode), total time in bed, sleep latency (time taken to fall asleep), wake after sleep onset (the total minutes scored as "wake" after sleep onset and before final awakening and sleep efficiency (the percentage of the total time patient spent in bed and was asleep; calculated as the total sleep time divided by time spent in bed) [9, 10, 11]. The purpose of this study is to describe the effect of supplementation of vitamin B12 in the form of cyanocobalamin on these nocturnal sleep parameters in healthy adults. Cyanocobalamin was selected, as it is the more common supplementation form of vitamin B12 [12]. Most previous studies have assessed sleep and vitamin B12 level using questionnaires or other subjective measures. This study objectively measures sleep and vitamin B12 level using actigraphy and serum concentrations of vitamin B12 before and after supplementation.

## 2 Material and methods

### 2.1 Participants

This study protocol was reviewed and approved by The University of West Indies, Mona Ethics Committee (Study number ECP50 17/18). The sample population were staff members of the Faculty of Medical Sciences between ages 25 and 50 years, who were healthy (free of infectious diseases, gastrointestinal reabsorption disorders and diarrhea). Exclusion criteria included low serum vitamin B12 levels (<150 pmol/L), vegetarians, pregnant or breastfeeding women, participants with sleep disorders or on sleep medication, participants with chronic non-communicable diseases including hypertension, heart failure, diabetes mellitus and asthma/chronic obstructive pulmonary disease, and any staff member who was currently in an academic programme. Convenience sampling was used with a flyer that was circulated through the faculty mailing list to invite interested staff members to contact the research team by email. Eligible participants were informed of the study by the student researcher and provided with the informed consent form. Only those staff members returning a signed written informed consent form were included.

### 2.2 Study design

This quasi-experimental pre-test, post-test designed study was conducted over four weeks. Actigraphy data were collected across two pre-supplementation weeks and two weeks during supplementation with 3mg of vitamin B12 (cyanocobalamin).

### 2.3 Cyanocobalamin supplementation

For the study, the Sundown Brand of vitamin B12 was purchased from local pharmacies. Each tablet contains one mg of cyanocobalamin. Tablets were prepackaged for participant to take three tablets (3mg) per day for 14 days.

### 2.4 Actigraphy sleep-wake activity assessment

The actiwatch-2 device (*Respironics, Inc., PA 15668, USA*) contains a solid-state multidirectional piezoelectric accelerometer (sensitive to ≥0.025g), which serially records the degree and intensity of motion as sleep activity counts by a built-in device software [9] and measured sleep latency (SL), total sleep time (TST), wake-after-sleep onset (WASO), total time in bed (TIB) and sleep efficiency (SE) at an epoch of 60 s. Threshold for awakening was set at 40 counts per 60 s.

### 2.5 Procedure

Participants were recruited from the Faculty of Medical Sciences, University of the West Indies, Mona campus between November 2020 and May 2021. Once consented for the study, participants were visited by the researcher at their various departments to initiate data collection. Data was collected for four-weeks from each participant. All Covid-19 protocols such as social distancing, wearing of masks, sanitization of hands and actiwatches, were observed. Demographic information collected included age, gender and any medications being taken. On Monday of week one of the study, 2–5 mL of blood was collected by venipuncture in a Red Top BD Vacutainer tube by the student researcher assigned to the project, who is a certified medical technologist. Blood samples were taken to the Chemical Pathology Laboratory, Department of Pathology, University of the West Indies, for determination of serum vitamin B12 levels using a multi-channel Cobas 6000 analyzer (*Roche Diagnostics, USA*). The participants were advised of their vitamin B12 status at the end of week one. Any participant returning a deficient vitamin B12 level (<150 pmol/L) as set by the World Health Organization [13] was excluded from the study at this point and those presenting readings above this level were allowed to continue in the study. Participants were given the actiwatch-2 device to wear on their non-dominant wrist from Monday morning to Thursday morning, following which they were required to return the watch to the researchers. The weekly data were downloaded, and watches recharged before being returned to the participants.

On Thursday of week two, participants were given pre-packaged cyanocobalamin supplements to be taken daily (starting on Friday of week two) for 14 days (including weekends). Venipuncture sampling was repeated at the end of the study to determine post-supplementation vitamin B12 levels.

### 2.6 Sample size

This study used a non-probability sampling technique. Sample size calculations were done using data from a pilot study conducted in the Faculty of Medical Sciences Department in 2018. From the pilot study, the mean (and standard deviation) presupplement TST was 366.24 (88.1) minutes and post supplementation TST was 460.98 (186.41) minutes. The mean difference from this was 94.7 min with a p = 0.100. To obtain an α (type 1 error) of 5% and a β (type 2 error) of 20% (power of 80%), it was determined that a sample size of 10 participant would be adequate to detect a significant mean difference of 94.7 min in TST. Anticipating a 50% drop out, a sample size of 15 was used.

### 2.7 Data analysis

For actiwatch-2 readings, the average over the three consecutive days was used for the weekly values of SL, TST, WASO, TIB and SE. Data collected were analyzed using the Statistical Package for the Social Sciences (SPSS, version 22). Descriptive statistics were used to quantify the data and expressed as medians and interquartile ranges [IQR] or counts and percentages. Medians were compared using the Wilcoxon signed rank test for paired continuous variables and the Mann-Whitney U test for independent continuous variables. Friedman's Two-way analysis of variance was used to access significant differences in the sleep parameters across the four weeks and associations were examined using Spearman's rho correlation. For all analyses, a p value of <0.05 was considered significant.

## 3 Results

Fifteen participants were recruited for the study; however, one male participant was excluded because of non-compliance with supplementation requirements of the study. Table 1 shows the gender, age, and serum vitamin B12 levels of the fourteen participants in the study. Most of the participants were females (n = 9, 64.3%) and the median [IQR] age of the group was 37 [17] years. One male participant did not want to state his age but confirmed that he was within the 25–50 years age group. No participant reported being on any medication.

**Table 1:** Sample characteristics and serum vitamin B12 concentration according to gender.

Demographics	Total (n = 14)	Male (n = 5)	Female (n = 9)
**Gender, n (%)** *Female* *Male*	9 (64.3) 5 (35.7)		
**Median [IQR] Age/years**	37 [17]	35 [19]	37 [16)]
**Median [IQR] Vitamin B12 Serum Level/pmol/L**			
*Pre-supplementation (n = 14)*	335 [217]	303 [269]	351 [255]
*Post-Supplementation (n = 12*^a^*)*	961 [679]	698 [404]	1392 [606]#
***p* value**	0.020∗	0.043∗	0.018∗

p value for differences between groups was calculated using Wilcoxon signed-rank test for paired continuous variables and Mann-Whitney U for independent continuous variables.

a Post-supplementation vitamin B12 concentration missing for two females.

∗ Significant Wilcoxon signed-rank test within groups (2-tailed).

# Significant Mann-Whitney U test between males and females (p = 0.030), at p < 0.05 significance level.

### 3.1 Pre-supplementation and post-supplementation difference in serum vitamin B12 levels

Vitamin B12 serum concentration was assessed on the first and last days of study period for each participant. Pre-supplementation serum vitamin B12 values ranged between 169 and 695 pmol/L and was not significantly different between genders (p = 0.438), as assessed using the Mann-Whitney U test. Post-supplementation vitamin B12 concentration was not obtained for two females due to reagent unavailability. However, Wilcoxon signed-rank test revealed that there was a significant increase in median serum vitamin B12 levels when post-supplementation levels were compared to pre-supplementation levels (p = 0.020). This significant increase was seen for both males (p = 0.043) and females (p = 0.018). Additionally, post-supplementation vitamin B12 concentration was significantly higher for females than males (p = 0.030).

### 3.2 Actigraphy-measured sleep analysis

Spearman's rho showed no correlation between the actigraphy sleep parameters and serum vitamin B12 levels for the pre-supplementation weeks when the whole group was assessed. Similar lack of correlation was observed between the actigraphy sleep parameters and serum vitamin B12 levels for the post-supplementation weeks. Table 2 shows the median and IQR results for the sleep parameters for the four weeks of the study. For week four, actigraphy data was missing for one female participant. Friedman's two-way analysis of variance was used to compare the weekly median times for SL, WASO, TST, TIB and the percentage for SE. There was no significant difference between sleep parameters when compared across the four weeks of actigraphy assessment (p > 0.05).

**Table 2:** Sleep measurements for three consecutive days (Mondays to Thursdays) of each week using wrist actigraphy.

Sleep (minutes)	Pre-supplementation Weeks		Supplementation Weeks		p value
	Week 1 (n = 14)	Week 2 (n = 14)	Week 3 (n = 14)	Week 4 (n = 13)^a^	
**Actigraphy data**					
*SL*	17 [29]	10 [11]	8 [12]	9 [9]	0.301
*WASO*	53 [32]	45 [23]	51 [25]	47 [19]	0.406
*TST*	407 [95]	371 [109]	386 [139]	367 [63]	0.520
*TIB*	488 [123]	429 [98]	474 [140]	436 [93]	0.089
*SE*	82 [10]	83 [5]	84 [9]	83 [7]	0.557

p value for differences between weeks calculated using Friedman's Two-way analysis of variance for continuous variable. Figures represent median [IQR].

∗Significant differences at p < 0.05 significance level.

SL, sleep latency; WASO, wake after sleep onset; TST, total sleep time; TIB, total time in bed; SE, sleep efficiency percentage.

a Sleep data missing for 1 participant for week 4.

## 4 Discussions

This study investigated the relationship between serum vitamin B12 levels and sleep parameters in healthy adults. Sleep data were collected using actigraphy for two weeks before and two weeks during supplementation with cyanocobalamin (3mg/day). There was no association between the serum vitamin B12 levels and sleep parameters measured by actigraphy. Similar lack of relationship between vitamin B12 and sleep parameters were reported in a study involving 355 female students, using Pittsburgh Sleep Quality Questionnaire [5]. However, other studies have reported conflicting results [4, 22].

The 14-days supplementation period resulted in a significant increase in the serum vitamin B12 concentration for both males and females; with higher levels for females compared to males. While we did not assess the possible reasons for this difference, other studies have suggested gender-based differences in vitamin B12 related metabolism [14, 15].

Despite the significant elevation in vitamin B12 concentration, this intervention did not change the sleep parameters of total sleep time, time in bed, wake after sleep onset, sleep latency, and sleep efficiency over the study period. Our study is the first to assess the influence of vitamin B12 supplementation on sleep parameters measured using actigraphy in healthy adults and overall found no significant changes. This contradicts the previous reports of Mayer et al [6], which also used actigraphy. However, this previous study involved taking the average of two consecutive days of measurements compared to this current study which used three consecutive days; recent literature on this objective method of assessing sleep-wake activity supports more than two days of measurements for increased sensitivity [16, 17].

Cyanocobalamin is metabolized to methylcobalamin upon absorption from the intestinal tract. Of the two forms of vitamin B12, only methylcobalamin passes the blood brain barrier and is important for the synthesis of melatonin, a major hormone that regulates sleep [18, 19, 20]. Interestingly, animal studies which involved direct injection of both forms of vitamin B12 into the third ventricle of the brain provided some indication that cyanocobalamin has no direct effect on sleep [21]. For future studies of cyanocobalamin supplementation on sleep, consideration should be given to including an analysis of changes in melatonin concentrations. Future studies should also include a control group, screen for physical health, control for diet, alcohol use and exercise, and exclude persons with mental illnesses/depression and stress.

There were a few limitations including sampling method being non-randomized and absence of a placebo control group. We had challenges recruiting male participants and there was loss of week four information for one of the female participants.

## 5 Conclusions

Two weeks of cyanocobalamin supplementation (3mg/day) resulted in increased serum concentrations but did not significantly alter the sleep parameters of total sleep time, time in bed, wake after sleep onset, sleep latency, and sleep efficiency in healthy adults over the four-week study period.

## Declarations

### Author contribution statement

Maxine Gossell-Williams and Peta-Gaye Thomas-Brown: Conceived and designed the experiments; Analyzed and interpreted the data; Contributed reagents, materials, analysis tools or data; Wrote the paper.

Paula Dawson: Conceived and designed the experiments; Contributed reagents, materials, analysis tools or data; Wrote the paper.

Toshiba Channer-Wallen: Conceived and designed the experiments; Performed the experiments; Contributed reagents, materials, analysis tools or data; Wrote the paper.

### Funding statement

This research did not receive any specific grant from funding agencies in the public, commercial, or not-for-profit sectors.

### Data availability statement

Data will be made available on request.

### Declaration of interests statement

The authors declare no conflict of interest.

### Additional information

No additional information is available for this paper.
